# A rapid identification technique for drug-resistant *Mycobacterium tuberculosis* isolates using mismatch specific cleavage enzyme

**DOI:** 10.6026/97320630014404

**Published:** 2018-07-31

**Authors:** Luis Jaramillo, David Tarazona, Kelly Levano, Marco Galarza, Omar Caceres, Maximilian Becker, Heinner Guio

**Affiliations:** 1 Laboratorio de Biotecnologia y Biologia Molecular, Centro Nacional de Salud Publica, Instituto Nacional de Salud, Lima, Peru

**Keywords:** *Mycobacterium tuberculosis*, MDR, XDR, Drug resistance, mismatch, cleavage

## Abstract

The emergence of multidrug-resistant tuberculosis (MDR-TB) and extensively drug-resistant tuberculosis (XDR-TB) strains is a major health problem for high Tuberculosis (TB) incidence countries. Therefore, it is of interest to identify antibiotic resistant bacteria by mismatch detection using DNA hybridization. We generated PCR products for five genes (rpoB, inhA, katG, gyrA and rrs) associated with drug resistance TB from MDR and XDR Mycobacterium tuberculosis (MTB) DNA samples. These were hybridized to PCR products from MTB H37Rv (pansusceptible laboratory strain) to generate DNA hetero-duplex products, which was digested by Detection Enzyme (GeneArt Genomic Cleavage Detection Kit) and visualized by agarose gel electrophoresis. Results show different bands with sizes of 400 bp and 288 bp (rpoB), 280 bp (inhA), 310 bp (katG), 461 bp (gyrA) and 427 bp (rrs) suggesting mutations in DNA heteroduplex for each gene. Detection Enzyme specifically cleaves DNA hetero-duplex with mismatch. The technique helps in the improved detection of MDR (mutations in rpoB, inhA and katG) and XDR (mutations in rpoB, inhA katG, gyrA and rrs) MTB strains. Moreover, the technique is customized without expensive specialized equipment to detect mutations. It is also fast, efficient and easy to implement in standard molecular biology laboratories.

## Background

In 2016, there were an estimated 10.4 million incident cases of TB (range, 8.8 million to 12.2 million) [1]. The estimated global burden of MDR-TB is 500 000 patients and approximately half of MDR-TB new cases are found in China, India and Russia [2]. Peru accounts for 14% TB patients and 35% of MDR patients in America [3]. Different studies indicate that mutations in rpoB, inhA, katG, gyrA and rrs MTB genes confer drug resistance to rifampicin (RIF), isoniazid (INH), fluoroquinolone (FLQ) and aminoglycosides (AMG), respectively [4]. In MDR-TB, bacterium is resistant to the main two first-line antibiotics (INH and RIF). rpoB mutations are restricted to an 81bp hot spot region resulting in amino acid substitutions responsible for a minimum of 95% resistance to RIF [5]. inhA and katG mutations correspond to 20% and 70% of INH resistance, respectively. XDR-TB causes drug resistance to RIF, INH, FLQ and at least one of the aminoglycosides such as capreomycin (CAP), kanamycin (KAN) or amikacin (AMK) [6]. gyrA mutations account for more than 90% of drug resistance to FLQs. Mutations in rrs gene correlate phenotypically with high levels of resistance to KAN, AMK and CAP [6] in specific regions.

Several methods are available to detect drug resistance in TB. These include RFLP-PCR [7], multi-allele specific PCR [8], realtime PCR by high resolution melting (PCR-HRM) [9], and GenoType MTBDRplus [10]. However, these methods have shown limited application in low-resource settings due to host cost with specialized equipment and trained staff [7, 10]. Therefore, there is a need for a fast, efficient and easy to implement method. It is of interest to identify antibiotic resistant bacteria by mismatch mutation detections in gene fragments associated to drug resistance in MTB samples.

## Methodology

We used DNAs isolated from MTB culture samples. Samples were confirmed for their drug-resistance using drug susceptibility testing (DST) for first- and second-line anti-tuberculosis drugs using agar proportion method [4]. The five steps used in sample processing are outlined below.

### Genomic DNA extraction

Sixteen microbial DNA samples were extracted using the PureLink™ Genomic DNA kit (ThermoFisher Scientific, USA), using the procedure described by the manufacturer.

### Primer design

PCR primers were designed for each target region (Table 1) using Primer 3 software [11]for amplification of polymorphic regions in rpoB, inhA, katG, gyrA and rrs genes.

### DNA fragments amplification

Polymorphic regions that have been associated to drug resistance in each gene were selected using known literature [7, 12]. Five regions were amplified by PCR: DNA-rpoB, DNA-inhA, DNAKatG, DNA-gyrA and DNA-rrs in each one of 15 DNA samples.

### Hybridization with DNA reference

PCR fragments of five genes (rpoB, inhA, katG, gyrA and rrs) from M. tuberculosis H37Rv (pan susceptible laboratory strain) was used to hybridize with PCR products obtained from the samples. The hybridization between DNA wildtype (H37Rv) and DNA mutant generated a hetero-duplex DNA. Hetero-duplex DNA was digested using Detection Enzyme of GeneArt Genomic Cleavage Detection Kit (ThermoFisher Scientific, USA), using the procedure described by the manufacturer. The enzyme recognizes the presence of mismatch. The cleaved DNA heteroduplex generated different DNA fragments size according to position mutation.

### Fragment analysis

Different band sizes of genes associated with drug resistance were visualized by agarose gel electrophoresis using a gel documentation system (Chemidoc XRS, Biorad, USA).

## Results & Discussion

**Figure 1:**
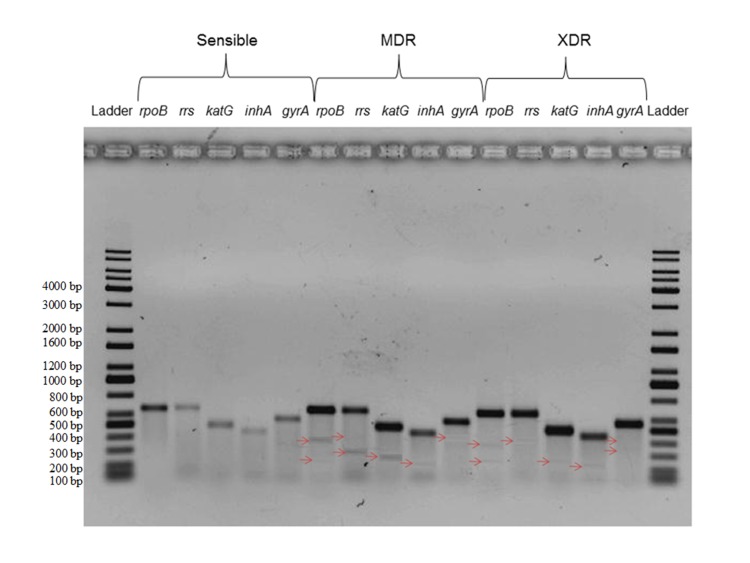
Five genetic fragments hybridization related to genotypic resistance to antituberculosis drugs using mismatch-specific DNA. Ladder: molecular weight marker (Kapa DNA ladder). Note: -> Fragments only in drug resistance bacteria.

Amplified fragments of rpoB, katG, gyrA, inhA and rrs genes were obtained using 10 designed primers given in Table 1. Gel electrophoresis band size patterns of sensitive M. tuberculosis, MDR and XDR are showed in Figure 1. We show different types of group fragments with positive mutations for drug resistance. In previous studies, fragment amplification was described as a powerful tool [13, 14]. We standardized primers and the annealing of PCR fragments with and without indels to form heteroduplex DNA.

We determined the presence of mutations associated with drug resistance according to the presence of different DNA fragment sizes providing a positive diagnosis for drug resistance (Figure 1). Results show that we can determine the presence of mutations revealed in DNA fragments: 400 bp and 288 bp (rpoB), 280 bp (inhA), 310 bp (katG), 461 bp (gyrA) and 427 bp (rrs) (Table 2).

There are advantages of mismatch cleavage endonucleaseI compared with other traditional mutation detection methods like single strand conformation polymorphism analysis, denaturing high-performance liquid chromatography, and heteroduplex analysis [15]. There is detection of all types of base substitution and insertion/deletion mismatches; cleavages of the fragments provide information about the location of mutation and multiple cleavage products indicate the presence of more than one variant [15]. According to our study and other's experience [16], endonucleaseI is a much better mismatch cleavage enzyme than phage resolvases because the latter produce many nonspecific bands, are size-limited, and additional experience is required for experiment assessment [17, 18, 19, 20]. We demonstrated the utility of GeneArt Genomic Cleavage Detection Kit to detect mismatch in hetero-duplex DNA associated to drug resistance in MTB.

## Conclusion

We hybridized five DNA fragments from M. tuberculosis samples to detect mutations in genes associated with drug resistance. The technique detects the presence of mutations in DNA fragments in MDR and XDR-TB. This method does not require expensive specialized equipment. It is also is fast, efficient and easy to implement in standard molecular biology laboratories.

## Author Contributions

All authors read and approved the final manuscript.

## Conflict of interest

The authors declare no conflicts of interest.

## Figures and Tables

**Table 1:** Primers designed to amplify rpoB, inhA, katG, gyrA and rrs fragments of Mycobacterium tuberculosis.

Gene	Primer	Sequence (5' - 3')	Size (pb)	Annealing Temp. (°C)
rpoB	1F	GGTGCCGGTGGAAACCGACGACA	650	
	1R	GCCCCCGTCAATTCTAGTCCACCTCAGACGA		
rrs	2F	TCGTCTGAGGTGGACTAGAATTGACGGGGGC	630	
	2R	CATACGAGCTCTTCTCTAGAAGGTGATCCAGCCGCACCTT		
katG	3F	AAGGTGCGGCTGGATCACCTTCTAGAGAAGAGCTCGTATG	500	60
	3R	ATTGCGCAGCAGGTATGTTCTAGAGACGAGGTCGTGGCTGA		
inhA	4F	TCAGCCACGACCTCGTCTCTAGAACATACCTGCTGCGCAAT	450	
	4R	GGTGGTAGTTGCCCATTCTAGATCACATTCGACGCCAAAC		
gyrA	5F	GTTTGGCGTCGAATGTGATCTAGAATGGGCAACTACCACC	550	
	5R	CTTCTACCTCAACAACTCCGCGC		

**Table 2:** Mismatch-specific DNA frangment sizes for drug resistance bacterium by electrophoresis gel analysis using Chemidoc XRS Software (Biorad, USA).

Gene	Fragment sizes (bp)
rpoB	400 - 288
inhA	280
katG	310
gyrA	461 - (360 in XDR)
rrs	427 - (330 in MDR)
